# RESEARCH CAN CHANGE PROGRAMS AND POLICY

**DOI:** 10.1093/geroni/igz038.862

**Published:** 2019-11-08

**Authors:** Lori Gerhard

**Affiliations:** U.S. Administration for Community Living, Washington, District of Columbia, United States

## Abstract

In addition to increasing knowledge, research is meant to improve practice and policy. The papers presented in this symposium draw from the experiences and insights of actual participants and their caregivers in major government-sponsored program options for people with disabilities wanting to remain in the community. Many of these programs are administered by our Administration for Community Living. For all, ACL serves as a main source of information for people in the community. These papers give us first-hand knowledge of what participants like and what they want improved. They give us guidance on how consumers define quality; the results can guide efforts to improve program design and the training of support brokers and representatives who assist people who want to manage their own supports and services. I will give a few examples starting with the paper on the Veterans-Directed Care Program and drawing ideas from the other papers.

